# 513 Lovenox Titration in Burn Patients: Does a One Size Fits All Model Work?

**DOI:** 10.1093/jbcr/irad045.110

**Published:** 2023-05-15

**Authors:** Anita Wamakima, Jeffrey Anderson, Lisa Rae, Jessica Hyman, Devon Romano, Huaqing Zhao, Xiaoning Lu

**Affiliations:** Temple University, Philadelphia, Pennsylvania; Temple University, Philadelphia, Pennsylvania; Temple University, Philadelphia, Pennsylvania; Temple University, Philadelphia, Pennsylvania; Temple University, Philadelphia, Pennsylvania; Temple University, Philadelphia, Pennsylvania; Temple University, Philadelphia, Pennsylvania

## Abstract

**Introduction:**

Venous thromboembolism (VTE) presents a significant morbidity to burn injured patients. The Western Trauma Association (WTA) and American Association of Trauma Surgeons (AAST) have recently developed guidelines for VTE prophylaxis dosing in trauma patients in which doses are adjusted based on anti-Xa levels. We sought to determine whether adopting these guidelines would lead to improved VTE prophylaxis in patients admitted to our burn service.

**Methods:**

We initiated a protocol at our American Burn Association verified burn center of titrating VTE prophylaxis for our patients based on anti-Xa levels consistent with the WTA and AAST guidelines. We performed a retrospective chart review of all patients in which anti-Xa levels were measured between May and October 2022. Chi square, Fisher's exact, and ANOVA were used to analyze the data.

**Results:**

A total of thirty six patients met our inclusion criteria. Age (49 [35.0, 61.0]), BMI (26.7 [6.8]), gender (67% male), and percent total body surface area (4.5 [2.0, 10.5]) were not statistically significant. Patients with a final lovenox dose of 30mg twice daily required a significantly lower dose adjustment than patients with a final lovenox dose of greater than 30mg (5.1 vs 7.9, p=0.0003). While not statistically significant, there was a trend for patients with a final lovenox dose of greater than 30mg twice daily to have larger percent TBSA affected (6.0 vs 13.6, p = 0.079). When comparing patients whose final dose of lovenox was less than, equal to, or greater than their initial dose, there was a significant difference in dose adjustment between patients whose lovenox had to be decreased as opposed to those who had to be increased (10.0 vs 15.8, p< 0.0001). None of our patients suffered a VTE event over the study period.

**Conclusions:**

A “one size fits all” approach to VTE prophylaxis in burn patients may lead to inappropriate prophylaxis. Our study demonstrates that titration of VTE prophylaxis with lovenox using anti-Xa levels in the burn population is feasible and that patients with burns may require individualized dosing for appropriate prophylaxis.

**Applicability of Research to Practice:**

Our research demonstrates that titration of VTE prophylaxis in a burn population may be more appropriate than the traditional “one size fits all” approach.

